# mTOR signaling mediates ILC3-driven immunopathology

**DOI:** 10.1038/s41385-021-00432-4

**Published:** 2021-08-02

**Authors:** Claudia Teufel, Edit Horvath, Annick Peter, Caner Ercan, Salvatore Piscuoglio, Michael N. Hall, Daniela Finke, Frank M. Lehmann

**Affiliations:** 1 Department of Biomedicine and University Children’s Hospital of Basel, University of Basel, 4058 Basel, Switzerland; 2 Department of Biomedicine, University of Basel, 4056 Basel, Switzerland; 3 Institute of Medical Genetics and Pathology, University Hospital Basel, 4056 Basel, Switzerland; 4 Biozentrum, University of Basel, 4056 Basel, Switzerland

## Abstract

Innate lymphoid cells (ILCs) have a protective immune function at mucosal tissues but can also contribute to immunopathology. Previous work has shown that the serine/threonine kinase mammalian target of rapamycin complex 1 (mTORC1) is involved in generating protective ILC3 cytokine responses during bacterial infection. However, whether mTORC1 also regulates IFN-γ-mediated immunopathology has not been investigated. In addition, the role of mTORC2 in ILC3s is unknown. Using mice specifically defective for either mTORC1 or mTORC2 in ILC3s, we show that both mTOR complexes regulate the maintenance of ILC3s at steady state and pathological immune response during colitis. mTORC1 and to a lesser extend mTORC2 promote the proliferation of ILC3s in the small intestine. Upon activation, intestinal ILC3s produce less IFN-γ in the absence of mTOR signaling. During colitis, loss of both mTOR complexes in colonic ILC3s results in the reduced production of inflammatory mediators, recruitment of neutrophils and immunopathology. Similarly, treatment with rapamycin after colitis induction ameliorates the disease. Collectively, our data show a critical role for both mTOR complexes in controlling ILC3 cell numbers and ILC3-driven inflammation in the intestine.

## Introduction

Innate lymphoid cells (ILCs) are tissue-resident cells with protective but also inflammatory immune functions. ILCs can be grouped into three subsets^1^. Group 1 ILCs (ILC1s) depend on the transcription factor T-box expressed in T cells (T-bet) and secrete T helper (Th)1 cytokines like interferon (IFN)-γ. ILC2s rely on GATA3 and secrete Th2 cytokines. ILC3s express and depend on the transcription factor RAR-related orphan receptor gamma t (RORγt)^2,3^ and can be subdivided into natural cytotoxicity receptor (NCR)^+^ and NCR^−^ cells^1^. Similar to Th22 and Th17 cells ILC3s secrete interleukin (IL)−22 and IL-17 upon activation^4–6^.

Both cytokines are found to be protective against intestinal pathogens like *Citrobacter rodentium* and contribute to the maintenance of gut tissue homeostasis^7–12^. Conversely, an inappropriate activation of ILC3s plays a role in intestinal immunopathology. In particular, the enhanced secretion of IFN-γ, CSF2, and IL-17 by ILC3s is detrimental in various colitis mouse models^13–17^. In recombination-activating gene 2 (*Rag2)*-deficient mice treated with α-CD40 antibodies (Abs) to elicit colitis, the release of IL-23 by myeloid cells activates colonic ILC3s to switch to pro-inflammatory IFN-γ production, thereby promoting the disease^13,15,16,18^.

mTOR exists in two different complexes, mTORC1 and mTORC2^19,20^, as they encompass different scaffolding proteins. RAPTOR (regulatory-associated protein of mTOR) is found in mTORC1 whereas RICTOR (rapamycin-insensitive companion of mTOR) is part of mTORC2^19,20^. Both molecules are essential for the formation and function of the respective complex. mTORC1 promotes protective immune responses of ILC3s against *C. rodentium* infection^21^.

The extent to which mTOR signaling determines the activation-induced switch of ILC3 to IFN-γ production and immunopathology is unknown. Moreover, the role of mTORC2 for ILC3 cytokine release remains unprobed. In this study, we demonstrate that mTORC1 and mTORC2 differentially regulate ILC3s under steady state and activation conditions. Using mice with mTOR deficiency in ILC3s on *Rag2*^*−/−*^ background we show that IFN-γ release by ILCs and recruitment of IFN-γ-producing inflammatory neutrophils is impaired during colitis. Similarly, treatment of mice with rapamycin after colitis induction ameliorates immunopathology. These results reveal a novel role for mTOR in regulating IFN-γ-driven immunopathology by ILC3s.

## Results

### mTOR signaling regulates ILC3 cellularity in the small intestine

**Fig. 1:**
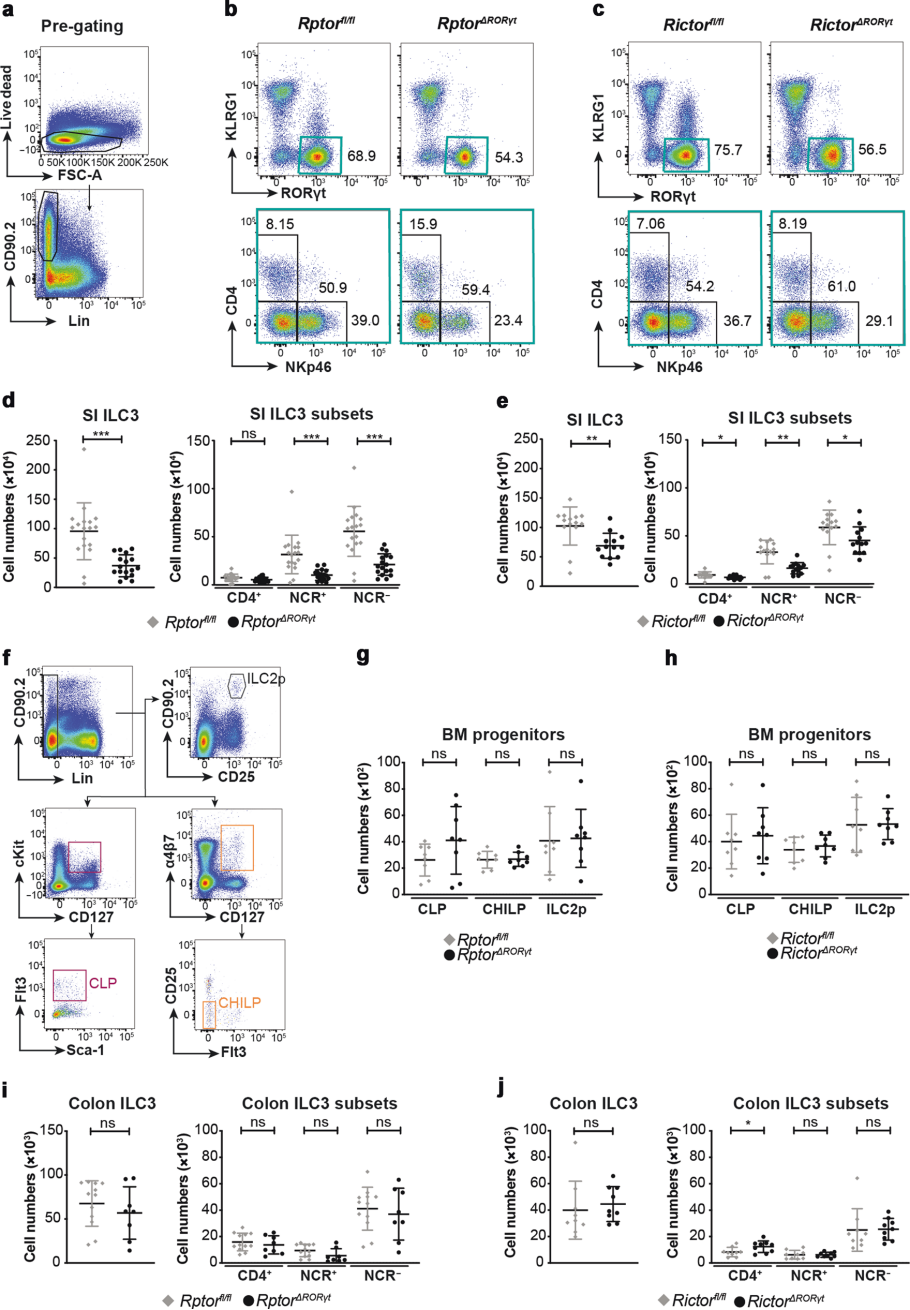
ILC3 subsets in *Rag2*^*−/−*^ mice depend on mTORC1 and mTORC2 signaling in vivo. Cells were isolated from SI LP of *Rptor*^*ΔRORγt*^ (**b**, **d**) or *Rictor*^*ΔRORγt*^ (**c**, **e**) mice and age-matched littermates. **a** Pre-gating for ILCs. Lin: CD3, CD8, CD11c, CD19, B220, Gr-1, TCRβ, TCRγδ, and Ter-119. **b**, **c** Exemplary dot plots of ILC3 subsets in the SI. The ILC3 subset (blue-marked gate, upper panel) was separated into CD4^+^, NCR^−^, and NCR^+^ ILC3 subsets (lower panel). **d**, **e** Total ILC3 numbers and numbers of CD4^+^, NCR^−^, and NCR^+^ ILC3s in the SI. *n* = 13–18, 6–8 independent experiments. **f**–**h** Cells were isolated from the tibia and femur of one hind leg of *Rptor*^*ΔRORγt*^ (**g**) or *Rictor*^*ΔRORγt*^ (**h**) mice and age-matched littermates and analyzed for the number of common lymphoid progenitors (CLPs), common helper ILC progenitors (CHILPs), and ILC2 progenitors (ILC2ps). *n* = 7–8, 4 independent experiments. **f** Gating strategy for hematopoietic progenitors. Lin: CD3, CD8, CD11c, CD19, B220, Gr-1, NK1.1, TCRβ, TCRγδ, and Ter-119. **g**, **h** Total numbers of CLPs, CHILPs, and ILC2ps in indicated mouse strains. **i**, **j** Total ILC3 numbers and numbers of CD4^+^, NCR^−^ and NCR^+^ ILC3s in the cLP of *Rptor*^*ΔRORγt*^ (**i**) or *Rictor*^*ΔRORγt*^ (**j**) mice and age-matched littermates. *n* = 8–12, 4 independent experiments. ns not significant; *p ≤ 0.05; **p ≤ 0.01; ***p ≤ 0.001, calculated with two-tailed unpaired Student’s *t* test or Mann–Whitney U test. Bars represent mean ± SD.

To study the function of mTORC1 and mTORC2-mediated signaling in ILC3s, mice with conditional deletion of *Rptor* or *Rictor* in ILC3s were generated and backcrossed to *Rag2*^*−/−*^ mice (named *Rptor*^*ΔRORγt*^ and *Rictor*^ΔRORγt^, for breeding details see “Material and Methods” section). The deletion of *Rptor* resulted in a reduction of NCR^+^ and NCR^−^ ILC3s in the small intestine (SI) at steady state (Fig. 1a, b, d and Supplementary Fig. 1a), as previously noted^21^. In addition, a lack of mTORC2 expression in ILC3s reduced the cellularity of SI NCR^+^, NCR^−^, and CD4^+^ ILC3 (Fig. 1a, c, e and Supplementary Fig. 1b). A small subset of KLRG1^+^RORγt^+^ ILCs was also reduced in the SI of both knockout mice (Supplementary Fig. 1c, d). This subset has been reported to express NKp46^+^ and to depend on T cell factor 1 (TCF-1; encoded by *Tcf7*)^22^. Of note, the reduced number of ILC3s was not a result of defective ILC progenitor development, as common lymphoid progenitor cell (CLP), common helper ILC progenitor (CHILP), and ILC2 progenitor (ILC2p) were normal in the bone marrow (BM) of both KO mice (Fig. 1f–h).

In contrast to SI ILC3s, colonic ILC3 numbers were normal in both mutant mouse strains, except for a small increase of CD4^+^ ILC3 numbers in the colon of *Rictor*^*ΔRORγt*^ mice (Fig. 1i, j, Supplementary Fig. 1e, f). The observation that the deletion of mTOR did not affect the majority of colonic ILC3s could be explained by a low mTORC expression or activation in colonic ILC3s. Indeed, colonic ILC3s showed a significantly lower phosphorylation of mTORC1 and mTORC2 targets S6 and AKT as compared to SI ILC3s (Supplementary Fig. 1g). Thus, mTOR signaling is dispensable for maintaining colonic ILC3 cellularity.

### mTOR promotes the expansion of intestinal ILC3s after bone marrow transplantation

**Fig. 2:**
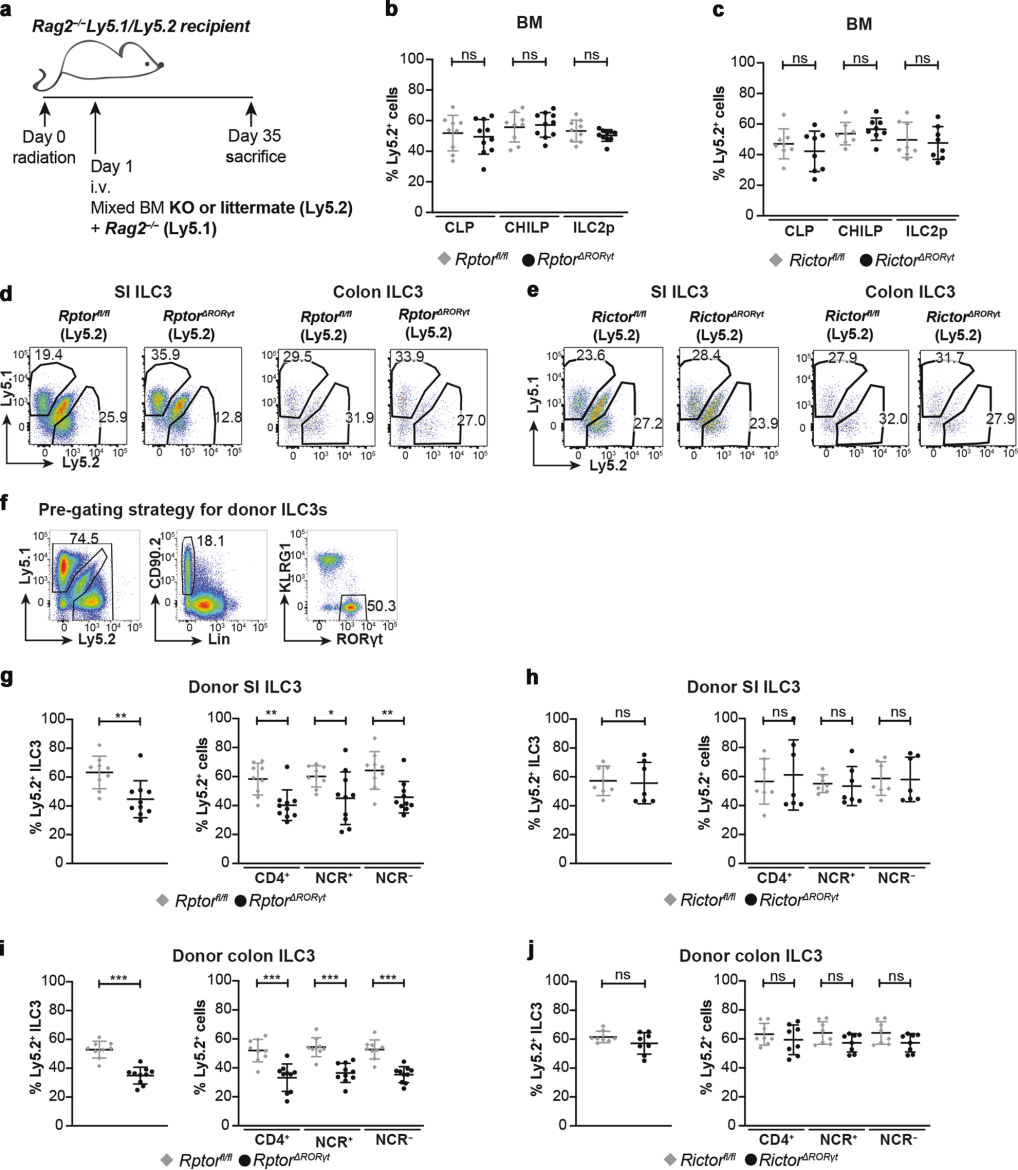
Competitive reconstitution of ILC3s in bone marrow chimeras depends on mTOR signaling. **a** Bone marrow chimera model: BM from *Rag2*^*−/−*^*Ly5.1*^*+*^ mice was mixed in a 1:1 ratio with BM from either *Rptor*^*ΔRORγt*^ mice (**b**, **e**, **g**, **i**), *Rictor*^*ΔRORγt*^ (**c**, **f**, **h**, **j**) mice or littermates, respectively. Five weeks after transplantation, mice were sacrificed. Cell were extracted from the SI LP, the cLP and the tibia and femur of one hind leg (BM). **b**, **c** Percentage of Ly5.2^+^Ly5.1^−^ CLPs, CHILPs, and ILC2ps in the BM was determined by flow cytometry. **d**, **e** Exemplary dot plots for Ly5.1 and Ly5.2 within ILC3 population. ILC3s were gated as Lin^−^CD90.2^+^KLRG1^−^RORγt^+^ (See Fig. 1). **f** Gating strategy for donor-derived ILCs for **g**–**j**. Lin: CD3, CD8, CD11c, CD19, B220, Gr-1, TCRβ, TCRγδ, and Ter-119. Percentage of Ly5.2^+^ cell within donor-derived ILC3s was determined in the SI (**g**, **h**) and colon (**i**, **j**). *n* = 7–10 mice from 2 to 3 independent experiments. ns not significant; *p ≤ 0.05; **p ≤ 0.01; ***p ≤ 0.001, calculated with two-tailed unpaired Student’s *t* test or Mann–Whitney U test. Bars represent mean ± SD.

To exclude indirect effects of mTOR-deficiency on ILC3s’ cellularity mixed BM chimeras were generated. BM cells from *Rptor*^ΔRORγt^, *Rictor*^*ΔRORγt*^ mice or litter controls (all Ly5.2) were mixed with *Rag2*^*−/−*^ BM cells (Ly5.1) in a 1:1 ratio and adoptively transferred in *Rag2*^*−/−*^ recipients (Ly5.1/Ly5.2) and analyzed 5 weeks later (Fig. 2a). Similar frequencies of donor-derived CLP, CHILP, and ILC2p were recovered from the BM of chimeric mice (Fig. 2b, c). In contrast, the frequencies of ILC3s from *Rptor*^*ΔRORγt*^ donors were reduced in the small and large intestine (Fig. 2d, f, g, i) revealing a competitive disadvantage of *Rptor*-deficient ILC3s under these experimental conditions. The deletion of *Rictor* in ILC3s did initially not affect their frequency in the SI of chimeric mice when probed 5 weeks after transfer (Fig. 2e, h, j) but resulted 10 weeks after transplantation in a moderately reduced percentage at this specific site (Supplementary Fig. 2a). Except for a modest reduction of colonic ILC1s, other ILC subsets, such as NK cells and ILC2s from *Rptor*^*ΔRORγt*^ and from *Rictor*^*ΔRORγt*^ donors were found in equal percentages as compared with litter controls in the SI and colon of recipient mice (Supplementary Fig. 2b, c). In aggregate, the loss of mTORC1 and to a lesser extent mTORC2 function compromises the expansion of mature intestinal ILC3s.

### mTOR signaling is required for IL-2-dependent proliferation of intestinal ILC3s

**Fig. 3:**
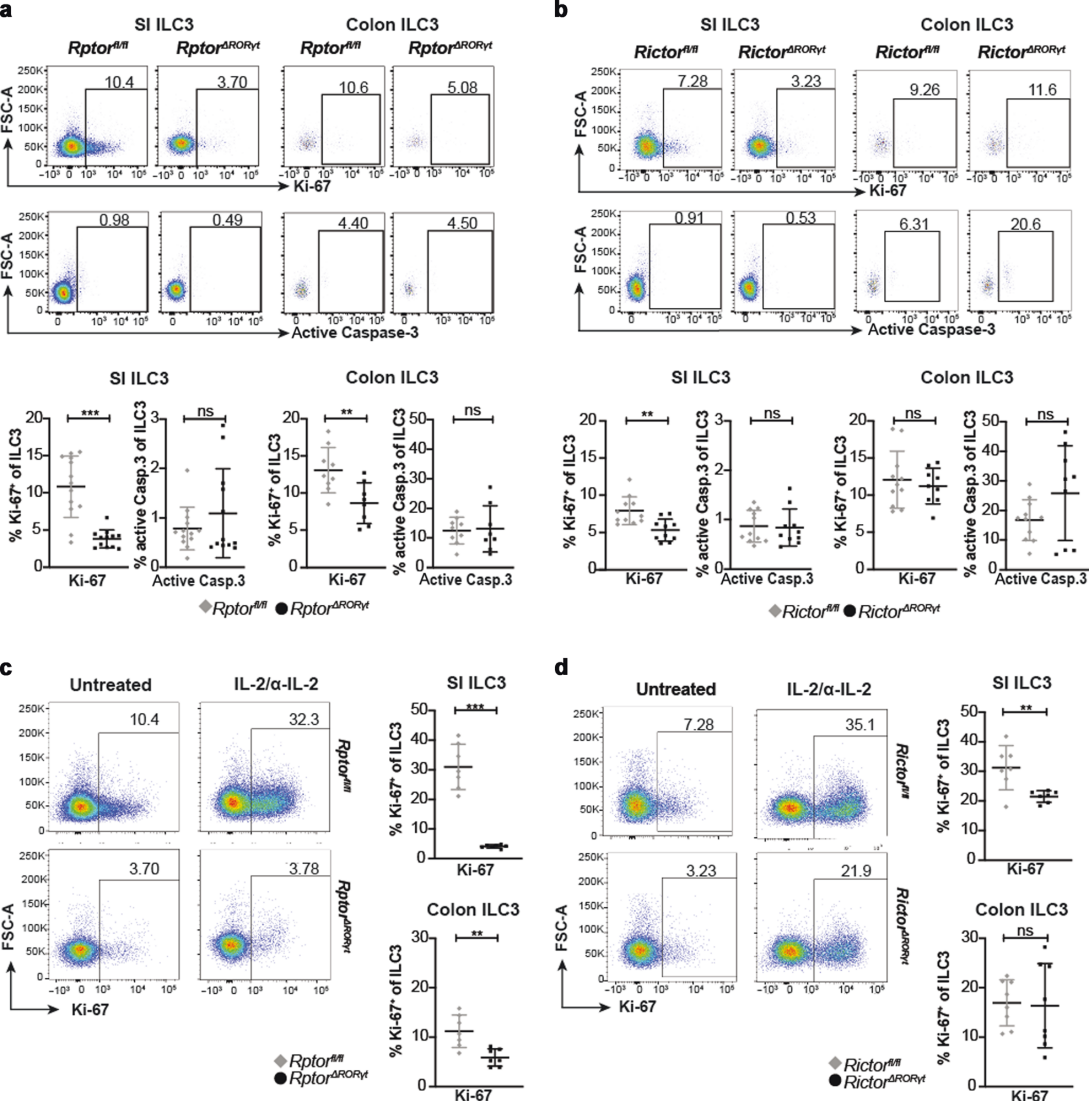
Disruption of mTOR signaling impairs ILC3 proliferation in vivo. Cells were isolated from the SI LP and cLP of *Rptor*^*ΔRORγt*^ (**a**) or *Rictor*^*ΔRORγt*^ (**b**) mice and age-matched littermates. **a**, **b** Exemplary dot plots of SI and colonic ILC3s. Percentage of Ki-67^+^ ILC3s and ILC3s positive for active Caspase-3 was determined by flow cytometry. ILC3s were gated as shown in Fig. 1. *n* = 10–12, 3–4 independent experiments. *Rptor*^*ΔRORγt*^ (**c**) or *Rictor*^*ΔRORγt*^ (**d**) mice and littermates were injected with one single dose of IL-2/α-IL-2 complex. After 1 day, cells from the SI LP and cLP were isolated and the percentage of Ki-67^+^ ILC3s was analyzed by flow cytometry. Exemplary dot plots from the SI of untreated and treated animals are shown on the left side. ILC3s were gated as shown in Fig. 1. *n* = 7 mice from 2 to 3 independent experiments. ns not significant; **p ≤ 0.01; ***p ≤ 0.001, calculated with two-tailed unpaired Student’s *t* test or Mann–Whitney U test. Bars represent mean ± SD.

We next determined Ki-67 expression and activated Caspase-3 in intestinal ILC3s to correlate the cells’ reduced cellularity to either diminished proliferation or impaired survival. Fewer Ki-67^+^ ILC3s were detected in *Rptor-*deficient mice in the SI and colon and in *Rictor*-deficient mice in the SI, whereas the frequencies were comparable in mutant and control animals when stained for activated Caspase-3 (Fig. 3a, b). These results indicated that mTOR regulated the cellularity of intestinal ILC3s via the promotion of cell proliferation.

IL-2 is essential for ILC3 and ILC2 proliferation^23–25^. We therefore tested the in vivo capacity of mTOR-proficient and deficient ILC3s to proliferate in response to IL-2. A third of litter control ILC3s located in both SI and colon proliferated within 24 h after exposure to IL-2 whereas *Rptor*-deficient ILC3s failed to expand (Fig. 3c). *Rictor*-deficient ILC3s responded to IL-2, albeit to a lesser extent than controls in the SI but normally in the colon (Fig. 3d). This was not a result of lower CD25 expression since we found comparable expression in colonic ILC3s of both groups and even higher expression in SI ILC3s of knockout mice (Supplementary Fig, 3a, b). Together, these data indicate that the proliferation of SI ILC3s depends on signals mediated by mTOR complexes.

### Activation-induced cytokine production by ILC3 depends on mTOR signaling

**Fig. 4:**
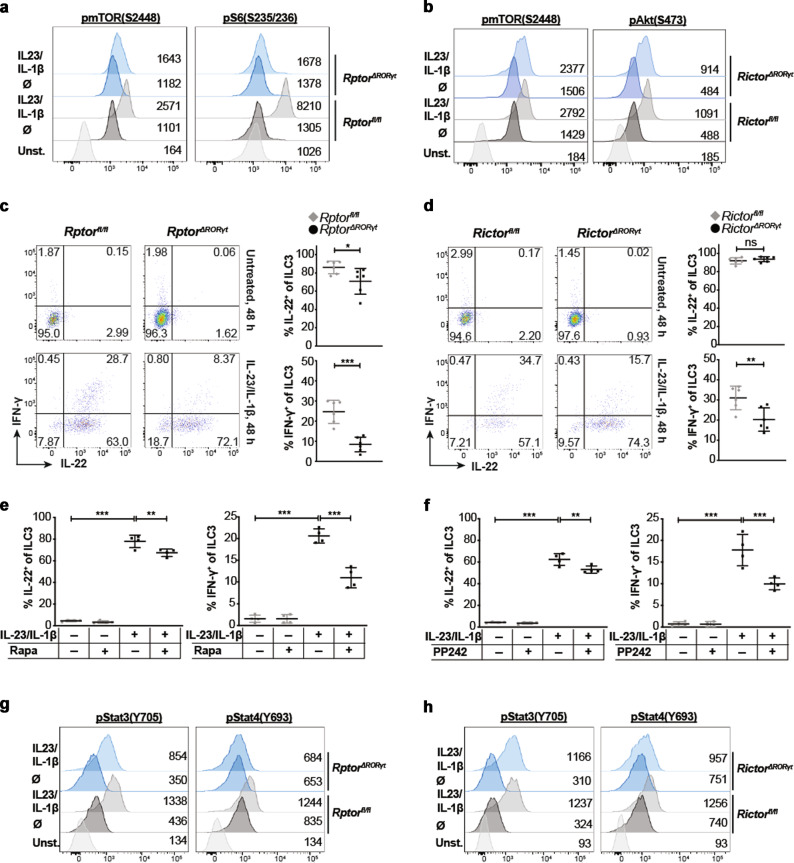
Loss of mTORC1 and mTORC2 signaling impairs activation-induced cytokine secretion. Lin^−^CD90.2^+^KLRG1^−^ ILC3s were sorted from the SI of *Rptor*^*ΔRORγt*^ (**a**, **c**) or *Rictor*^*ΔRORγt*^ (**b**, **d**) mice and control littermates and stimulated with 20 ng/ml IL-23 and IL-1β for 17 h (**a, b)** or for 2 days (**c, d**). The sorting strategy is depicted in Supplementary Fig. 5a. **a**, **b** Phosphorylation of mTOR, mTORC1-target site in S6 protein and mTORC2-target site in Akt was determined by phospho flow analysis. Depicted histograms (modal view) are representative of 4–6 independent experiments. Indicated values represent the geometric mean fluorescence intensity. **c**, **d** Intracellular IL-22 and IFN-γ was measured by flow cytometry. For each experiment, cells from 1 to 3 mice per group were pooled. *n* = 6 independent experiments. **e**, **f** Lin^−^CD90.2^+^KLRG1^−^ SI *WT* ILC3s were sorted and cultured with 20 ng/ml IL-23 and IL-1β or medium. Where indicated, 10 nM rapamycin (**e**) or 1 μM PP242 (**f**) was added. After 2 days, cells were stained for IL-22 and IFN-γ. For each experiment, cells from 8 to 10 mice were pooled. *n* = 4 independent experiments. Lin^−^CD90.2^+^KLRG1^−^ ILC3s were sorted from the SI of *Rptor*^*ΔRORγt*^ (**g**) or *Rictor*^*ΔRORγt*^ (**h**) mice and control littermates and cultured with 20 ng/ml IL-23 and IL-1β for 17 h. Phosphorylation of STAT3 and STAT4 was determined by phospho flow analysis. Depicted histograms (modal view) are representative of 3–6 independent experiments. Indicated values represent the geometric mean fluorescence intensity. Lin: CD3, CD8, CD11c, CD19, B220, Gr-1, NK1.1, TCRβ, TCRγδ, and Ter-119. ns not significant; *p ≤ 0.05; **p ≤ 0.01; ***p ≤ 0.001, calculated with two-tailed unpaired Student’s *t* test or one-way ANOVA with multiple comparison test (Bonferroni). Bars represent mean ± SD.

In the absence of RAPTOR, the activation of both mTOR and S6 kinase was reduced following exposure to IL-23 and IL-1β, both known to activate and stimulate the cytokine release of ILC3s (Fig. 4a and Supplementary Fig. S4a). Similarly, activated ILC3s isolated from *Rictor*^*ΔRORγt*^ mice displayed a reduced phosphorylation of mTOR, which correlated with a lower detection of phosphorylated Akt in these cells (Fig. 4b and Supplementary Fig. S4b).

We next probed whether cytokine responses of ILC3s upon stimulation with IL-23 and IL-1β were affected in mTOR-deficient mice. In contrast to controls where the large majority of SI ILC3s expressed IL-22 and 25% were positive for IFN-γ following stimulation, mTOR-deficient ILC3s displayed an impaired response to this activation (Fig. 4c, d). IFN-γ expression by ILC3s was threefold reduced in *Rptor*^*ΔRORγt*^ mice and 1.5-fold smaller in *Rictor*^*ΔRORγt*^ animals whereas the IL-22 response was only mildly diminished in *Rptor*- and unchanged in *Rictor*-deficient ILC3s. The observed decrease in IFN-γ production was confirmed by quantification of IFN-γ protein in the supernatant of in vitro activated SI ILC3s (Supplementary Fig. 4c–e). Finally, Rapamycin and PP242, effective inhibitors of the mTORC1 and mTORC1/2 pathway, respectively, impaired the IL-22 and even more pronounced IFN-γ responses of sorted SI ILC3s from wild-type (WT) mice (Fig. 4e, f and Supplementary Fig. 4c).

Activation of signal transducer and activator of transcription (STAT) 4 marked by phosphorylated tyrosine at position tyrosin (Y) 693 controls IFN-γ expression following IL-23 stimulation^26^. STAT4 phosphorylation was significantly reduced in activated ILC3s of *Rptor*^*ΔRORγt*^ and to a lesser extend in ILC3s of *Rictor*^*ΔRORγt*^ mice, which correlates with the quantitative reduction of ILC3s’ IFN-γ production in these mice (Fig. 4g, h and Supplementary Fig. 4f, g). The IL-22 production by ILC3s is normally controlled by IL-23 engaging STAT3 activation^9^. Phosphorylation of STAT3 at position Y705 was reduced in *Rptor*^*ΔRORγt*^ deficient mice but normal in *Rictor*^*ΔRORγt*^ mice (Fig. 4g, h and Supplementary Fig. 4f, g). We therefore conclude that both mTOR complexes are essential for the activation-induced production of IFN-γ, whereas only mTORC1 is relevant for the release of IL-22 upon IL-23 and IL-1β stimulation.

### mTOR deficiency in ILC3s ameliorates α-CD40-mediated colitis

**Fig. 5:**
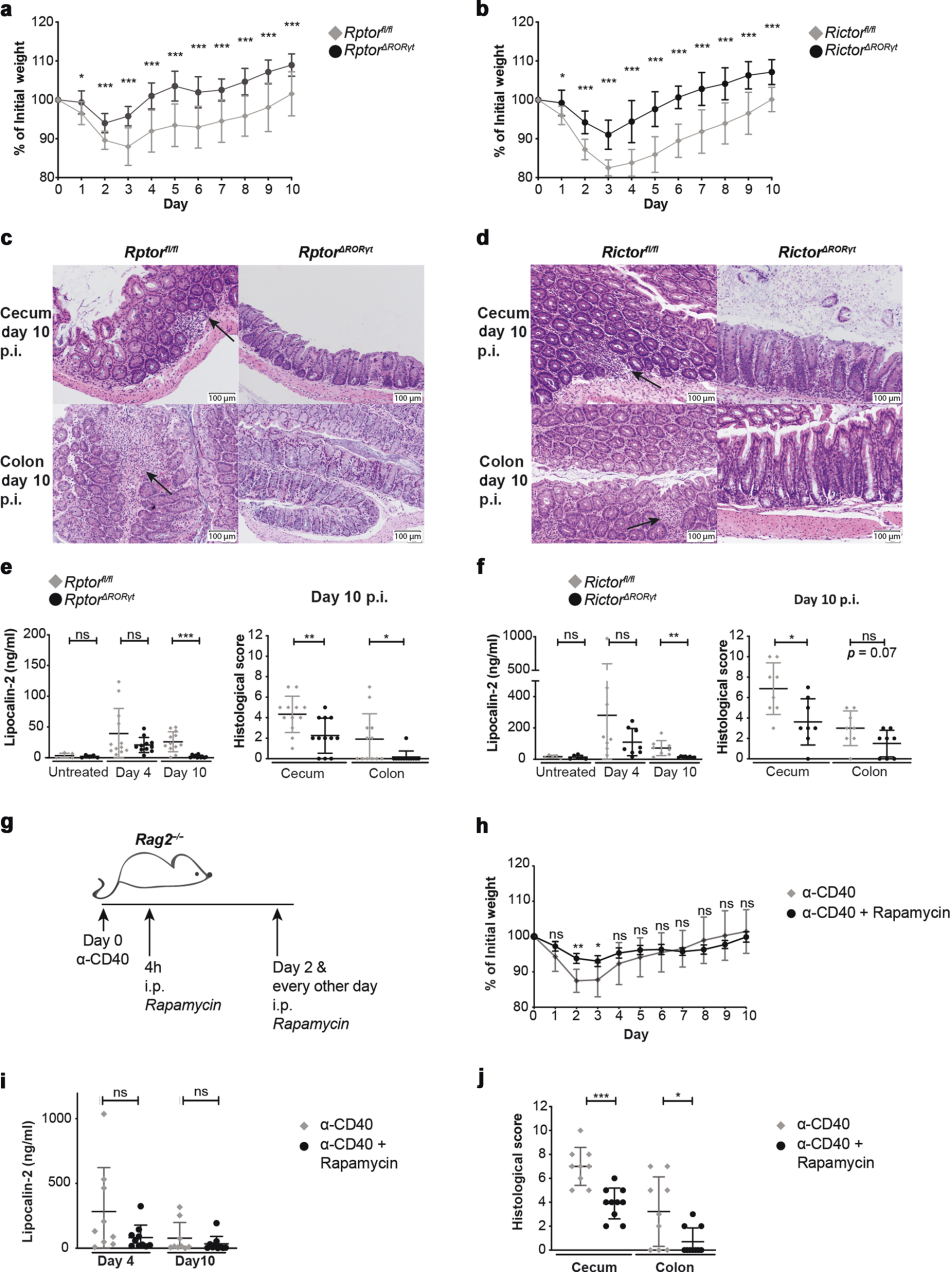
mTOR-deficiency in ILC3s protects from α-CD40-induced colitis. *Rptor*^*ΔRORγt*^ (**a**, **c**, **e**) or *Rictor*^*ΔRORγt*^ (**b**, **d**, **f**) mice and age-matched littermates were left untreated or were injected with a single dose of 140 μg α-CD40 Ab. *n* = 8–12 mice from 2 to 3 independent experiments. Weight was monitored daily (**a, b**). Mice were sacrificed 10 days post injection. The colon and the cecum were sectioned and stained with hematoxylin and eosin. Exemplary histology picture from the cecum and colon are depicted (**c**, **d**). Arrows indicate lymphocyte invasions. **e**, **f** At days 4 and 10, fecal Lipocalin-2 was determined from feces pellets diluted in PBS using ELISA. Histological colitis score was determined**. g** *Rag2*^*−/−*^ mice were left untreated or were injected with a single dose of 140 μg α-CD40 Ab. 4 h after α-CD40 Ab injection mice received 2.5 mg/kg rapamycin. Rapamycin injections were repeated at day 2 and every other day. **h** Weight was monitored daily. *n* = 8–10 mice. **i** At days 4 and 10, fecal Lipocalin-2 was determined from feces pellets diluted in PBS using ELISA. *n* = 9–10 mice of three independent experiments. **j** Mice were sacrificed 10 days post injection. The colon and the cecum were sectioned and stained with hematoxylin and eosin. Histological colitis score was determined. *n* = 7–10 mice of three independent experiments. ns not significant; *p ≤ 0.05; **p ≤ 0.01; ***p ≤ 0.001, calculated with two-tailed unpaired Student’s *t* test or Mann–Whitney U test. Bars represent mean ± SD.

In an experimental mouse model of colitis induced by α-CD40 Ab injection into *Rag2*-deficient mice it was shown that ILC3s contribute to the pathology by IL-23-driven release of IFN-γ^13,15,16^. As the absence of mTOR-mediated signaling reduced ILC3s’ capacity to secrete IFN-γ upon IL-23 stimulation, we next wished to probe whether *Rptor*^*ΔRORγt*^ and/or *Rictor*^*ΔRORγt*^ mice develop colitis following α-CD40 Ab treatment. In litter controls, one single injection with α-CD40 Ab resulted in weight loss, detection of epithelial-derived lipocalin-2 (LPC2) in the stool as a biomarker of tissue damage (at days 4 and 10) and histopathological signs of colitis at day 10 after Ab injection (For details of histological scoring see Material and Methods). In *Rptor*^*ΔRORγt*^ and *Rictor*^*ΔRORγt*^ mice weight loss, LPC2 expression and histopathological alterations in the cecum and colon mucosa were less severe than in controls (Fig. 5a–f). Confirming these findings, treatment of *Rag2*^*−/−*^ mice with Rapamycin after induction of α-CD40-induced colitis reduced weight loss on days 2 and 3 and the histological score in cecum and colon at day 10 (Fig. 5g–j).

**Fig. 6:**
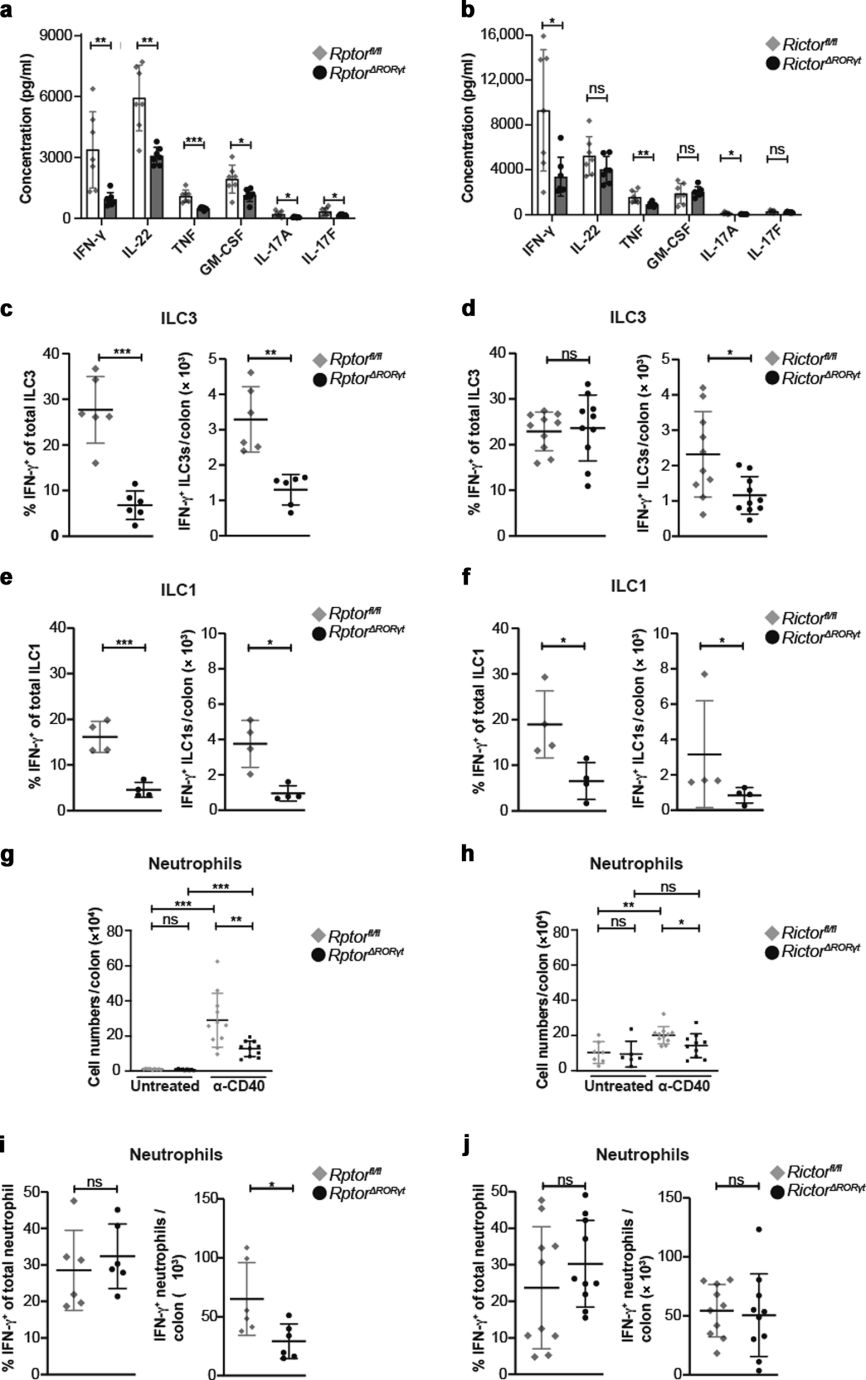
Impaired cytokine secretion and neutrophil infiltration in colonic tissue by deletion of either *Rptor* or *Rictor* in ILC3s. **a**, **c**, **e**, **g**, **i** *Rptor*^*ΔRORγt*^ or **b**, **d**, **f**, **h**, **j** *Rictor*^*ΔRORγt*^ mice and age-matched littermates were left untreated or received a single dose of 140 μg α-CD40 Ab. Mice were sacrificed 3 days post injection. **a**, **b** cLPs cells were isolated and cultured overnight. Concentrations of IFN-γ, IL-22, TNF, GM-CSF, IL-17A, and IL-17F in the supernatant were measured and normalized to 1 × 10^6^ cLPs cell count. *n* = 7 mice of 2–3 independent experiments. **c**–**f** cLP cells were cultured overnight. IFN-γ expression of ILC3s (**c**, **d**) and ILC1s (**e**, **f**) was analyzed by flow cytometry. ILC1 and ILC3s were gated as depicted in Fig. S5a. *n* = 6–10 mice from four independent experiments (ILC3) and *n* = 4 mice from two independent experiments (ILC1). **g**, **h** Colonic neutrophils were analyzed by flow cytometry at day 3 after α-CD40 Ab injection. Total neutrophil counts are depicted. Neutrophils were gated as depicted in Fig. S5b. *n* = 6–11 mice from 2 to 4 independent experiments. **i**, **j** cLP cells were cultured overnight and analyzed by flow cytometry. IFN-γ expression of neutrophils was analyzed by flow cytometry. Neutrophils were gated as depicted in Fig. S5a. *n* = 6–10 mice from four independent experiments ns not significant; *p ≤ 0.05; **p ≤ 0.01; ***p ≤ 0.001, calculated with two-tailed unpaired Student’s *t* test or Mann–Whitney U test. Bars represent mean ± SD.

*Rptor*^*ΔRORγt*^ and *Rictor*^*ΔRORγt*^ mice had normal numbers of colonic ILC3s (Fig. 1i, j), ILC1s or other ILC subsets (Supplementary Fig. S5a,b) indicating that the less severe colitis was not a result of a reduction of total ILC numbers. Instead, we hypothesized that the less pronounced colitis in *Rptor* and *Rictor*-deficient mice (Fig. 5a–f) was the consequence of a reduced local production of inflammatory cytokines in the gut. We therefore cultured lamina propria (LP) cells isolated from the colon of α-CD40-treated *Rptor*^*ΔRORγt*^ and *Rictor*^*ΔRORγt*^ mice at day 3 after colitis induction and analyzed their production of cytokines using LEGENDplex (Fig. 6a, b). Total LP cells from *Rptor*^*ΔRORγt*^ and *Rictor*^*ΔRORγt*^ mice produced less inflammatory cytokines, e.g. IFN-γ, TNF, IL-17A, as compared to litter control mice. In addition, the reduced intestinal immunopathology in *Rptor*^*ΔRORγt*^ and *Rictor*^*ΔRORγt*^ mice correlated with a reduction in colonic IFN-γ^+^ ILC3s and IFN-γ^+^ ILC1s (most likely ex-ILC3s) at day 3 upon colitis induction (Fig. 6c–f and Supplementary Fig. 5c).

ILC3 also control the recruitment of neutrophils to sites of inflammation indirectly via their release of IL-22, thus contributing to tissue damage^27^. In *Rptor*^*ΔRORγt*^ and *Rictor*^*ΔRORγt*^ mice with α-CD40-induced colitis, the number of tissue infiltrating neutrophils was reduced at day 3 compared to littermate controls (Fig. 6g, h and Supplementary Fig. 5d–f). Besides ILCs also neutrophils expressed IFN-γ during colitis. IFN-γ^+^ neutrophil cell numbers  were reduced in the colon of *Rptor*^*ΔRORγt*^ but not *Rictor*^*ΔRORγt*^ deficient mice (Fig. 6i, j). In contrast to recent publications about RORγt^+^ neutrophils^28,29^ no RORγt expression in intestinal neutrophils of *Rorγt*^*fm+*^ *Rag2*^*−/−*^ mice treated with or without α-CD40 Ab was detected (Supplementary Fig. 5g). In summary, we demonstrated that the deletion of mTOR in ILC3s reduced the release of inflammatory cytokines and the recruitment of neutrophils which correlated with limited tissue damage in an experimental model of colitis.

## Discussion

In the present study, we uncovered the role of mTORC1 and mTORC2 in regulating cellularity and inflammatory immune responses of ILC3s. Whereas conventional ILC progenitors were normal in *Rptor*^*ΔRORγt*^ and *Rictor*^*ΔRORγt*^ mice, ILC3s had a defect in expansion through proliferation. In addition, ILC3s from *Rptor*^*ΔRORγt*^ or *Rictor*^*ΔRORγt*^ mice were completely or partially resistant to stimulation with α-IL-2/IL-2 complexes, a strong inducer of proliferation^25,30,31^.

Two recent studies addressed the role of mTORC1 signaling for ILC3s^21,32^. In agreement with our results, Di Luccia noticed a reduction of SI ILC3s in mTORC1-deficient mice and an inhibitory effect of rapamycin on the proliferation of in vitro stimulated human ILCs and an ILC3-like cell line. Our data demonstrate that under steady state conditions, cell numbers of ILC3 were less dependent on mTORC1 and mTORC2 in the colon as compared to the SI. Analysis of mTOR targets revealed that mTORC1 and mTORC2 were less active in colonic as compared with SI ILC3s. It is likely that the concentration of several metabolites and cytokines that target mTOR phosphorylation in ILC3s varies between SI and colon^33,34^.

Previously, mTORC1 signaling was shown to be important for IL-22 release by ILC3s upon in vitro stimulation with IL-23/IL-1β^21,34^ and upon infection with C. rodentium^21^. In contrast, our data demonstrate that IL-22 production induced by IL-23/IL-1β was only moderately reduced in ILC3s from *Rptor*^*ΔRORγt*^ mice and normal in ILC3s from *Rictor*^*ΔRORγt*^ mice. Importantly, deletion of either mTORC1 or mTORC2 strongly impaired the IFN-γ-release by ILC3s after IL-23 and IL-1β stimulation. The reduced secretion of IFN-γ correlated with a reduced phosphorylation of STAT4, which is a transcriptional activator of IFN-γ in ILC3s^26^.

The intestinal immunopathology in the α-CD40 colitis model is driven by the release of IL-23 by myeloid cells^15,16,18^. As shown in Fig. 4a, b IL-23 activated both mTOR pathways in ILC3s upon in vitro stimulation. Hence, it can be assumed that both pathways are activated in the α-CD40-induced colitis model. The severe reduction of IFN-γ release together with a reduction of other cytokines (e.g. TNF, GM-CSF) upon colitis induction might explain why *Rptor*^*ΔRORγt*^ and *Rictor*^*ΔRORγt*^ mice were partially protected in an α-CD40-mediated colitis model^16–18^. Interestingly we observed a reduced release of IFN-γ by ILC3s and ILC1s, which can be explained by the differentiation of ILC3s into an ILC1 subset named “ex-ILC3s”^35^. Besides ILCs we noticed a reduced infiltration with IFN-γ-secreting neutrophils, which were described as an important source of IFN-γ during infection-induced colitis^36^.

Previously it has been shown that IL-22 induces the expression of neutrophil attracting chemokines in colonic lamina propria (cLP) during α-CD40-induced colitis^27^ and DSS-induced colitis^37^. In line with this, we found a reduced secretion of IL-22 in the colon of *Rptor*-deficient mice during colitis. In addition, we noticed a reduction of IL-17A and IL-17F in the colon which are also regulators for the mobilization of neutrophils^38,39^. Hence the reduced expression of such neutrophil-mobilizing cytokines by ILC3s in mTOR-deficient mice might be responsible for the reduced infiltration with neutrophils during α-CD40-induced colitis.

Interestingly, we noticed throughout our study that the deletion of *Rptor* or *Rictor* impaired similar biological functions of ILC3s, with a stronger effect of the deletion of *Rptor* on ILC3s’ cellularity and proliferation at steady state. The IFN-γ response of ILCs during colitis, however, was similarly reduced in both knockout strains. It is likely that both mTOR complexes cooperate in the regulation of the underlying signaling pathways that promote proliferation or IFN-γ responses by ILC3s. Positive and negative crosstalk between both mTOR complexes has been described^40,41^. Whether such positive or negative signals are relevant in the pathways regulating ILC3s needs to be analyzed in further experiments.

We observed a less pronounced weight loss in *Rictor*^*ΔRORγt*^ mice as compared to *Rptor*^*ΔRORγt*^ mice. This difference might be explained by the finding that IL-22, which is normal in *Rictor*^*ΔRORγt*^ but reduced in *Rptor*^*ΔRORγt*^ mice (Figs. 4c, d and 6a, b*)*, is involved in wasting disease during α-CD40-induced colitis^27^.

The results from our colitis model prompted us to test an mTORC1 inhibitor in this model. We found that rapamycin treatment after colitis induction ameliorated the disease in *Rag2*^*−/−*^ mice, pointing to a potential benefit for inflammatory bowel disease (IBD) patients of therapy with mTORC1 inhibitors. In support of this assumption, the treatment with rapamycin was beneficial for adult and pediatric IBD patients in two case studies^42,43^.

Collectively, our data shed light on the role of mTOR signaling regulating ILC3s numbers at steady state and inflammatory cytokine release during ILC3-driven intestinal immunopathology.

## Material and methods

### Antibodies

α-CD3ε (145-2C11), α-CD4 (RM4-5 or GK1.5), α-CD8α (53–6.7), α-CD11b (M1/70**)**, α-CD11c (N418), α-CD19 (6D5), α-CD45R (RA3-6B2, B220), α-CD90.2 (Thy1.2, 30-H12), α-Gr-1 (RB6-8C5, Ly-6G), α-TCR-β (H57-597), α-TCR-γδ (UC7-13D5), α-TER-119 (TER-119), α-NKp46 (29A1.4), α-NK1.1 (PK136), α-KLRG1 (2F1), α-p-STAT3 (Tyr705) (13A3-1), α-RORγt (AFKJS-9 or B2D), α-IFN-γ (XMG1.2), α-TNF (MP6-XT22), α-IL-22 (1H8PWSR), α-p-S6 (Ser235/236) (D57.2.2E), α-p-mTOR (Ser2448) (MRRBY), α-Ly-6G (1A8), α-Ly-6C (HK1.4), α-Ki-67 (B56), α-Eomesodermin (Dan11mag), α-CD45.1 (A20), α-CD45.2 (104), α-CD127 (IL-7Rα) (A7R34), α-CD117 (cKit) (2B8), α-active Caspase-3 (C92-605), α-CD25 (PC61), α-CD45 (30-F11), α-CD135 (FLT3) (A2F10), α-α4β7 (LPAM-1) (DATK32), α-T-bet (eBio4B10), α-Sca-1 (D7), α-STAT3 (Tyr705) (13a3-1), α-STAT4 (Tyr 693) (38/p-Stat4), and α-p-AKT(Ser473) (M89-61) were ordered either form BD Biosciences, Biolegend, eBioscience or Cell Signaling Technology. α-CD40 (FGK45.5) Ab was produced in B-cell hybridoma cells (kindly provided by Antonius Rolink) according to standard methods.

### Mice

Specific-pathogen-free animals were bred and maintained at the animal facilities of the Department of Biomedicine (University of Basel, Switzerland). The animal experiments received the approval of the Cantonal Veterinary Office of the city of Basel, Switzerland. Organ donors for in vitro experiments were 7–17 weeks of age. For in vivo experiments, age- and sex-matched animals were used. Animals were 8–11 weeks of age at the initiation of in vivo experiments.

*C57BL/6* WT and *Rag2*^*−/−*^ mice^44^ were purchased from Janvier Labs and Taconic, respectively. *RORc(γt)-Cre*^*tg*^ mice^45^ and *Rag2*^*−/−*^ *Ly5.1* were ordered from The Jackson Laboratory. *Rptor*^*fl/fl*^ and *Rictor*^*fl/fl*^ mice^46^ were kindly provided by M.N.H. (Biozentrum, University of Basel).

To generate *Rptor*^*ΔRORγt*^ mice, *RORc(γt)-Cre*^*tg*^ mice were crossed with *Rptor*^*fl/fl*^ mice and then bred to *Rag2*^*−/−*^ mice. To receive *Rictor*^*ΔRORγt*^ mice, *RORc(γt)-Cre*^*tg*^ mice were bred to *Rictor*^*fl/fl*^ mice and backcrossed to *Rag2*^−/−^ mice. In experiments with *Rptor*^*ΔRORγt*^ mice *and Rictor*^*ΔRORγt*^ mice, we used *Cre*^*−*^ littermates as controls. To generate *Rag2*^*−/−*^ *Ly5.1/Ly5.2* mice, *Rag2*^*−/−*^ mice were mated with *Rag2*^*−/−*^ *Ly5.1* mice. *Rorγt*^*fm+*^ mice were received by breeding *RORc(γt)-Cre*^*tg*^ mice to *Rosa26R*^*eYFP/+*^ mice^45,47^. *Rorγt*^*fm+*^ mice were backcrossed to *Rag2*^*−/−*^ mice.

### Cell isolation

For isolation of LP cells of the SI, the SI was dissected and mesenteric tissue was removed. The SI was opened longitudinally, incubated in 30 mM EDTA in calcium-/magnesium-free 1x PBS for 30 min and washed repeatedly in cold 1x PBS to remove epithelial cells, feces and mucus. The tissue was digested four times for 15 min at 37 °C in DMEM (Gibco) containing 0.025 mg/ml DNase I (Roche) and 1 mg/ml Collagenase D (Roche). Before and after each digestion step, the tissue was homogenized and washed with DMEM. Homogenate supernatant was collected through a 100 μM cell strainer and stored in DMEM supplemented with 5% fetal bovine serum (FBS) and 2 mM EDTA. Cell suspension was pelleted, resuspended in 40% of isotonic Percoll (GE Healthcare), underlaid with 80% isotonic Percoll and separated by gradient centrifugation (30 min, 20 °C, 1800 rpm, acceleration speed 4, deceleration speed 1). The cell interphase was collected, washed and used for direct staining or for sorting.

To isolate cLP cells, the colon was opened longitudinally, washed in 1x PBS, cut into pieces and washed four times on a shaker in 5 μM EDTA and 10 mM HEPES in 1x PBS for 20 min at 37 °C. The tissue was transferred into a gentleMACS™ C Tube (Miltenyi Biotec) filled with DMEM supplemented with 0.025 mg/ml DNase I, 1 mg/ml Collagenase D and 0.25 mg/ml Collagenase VIII (Roche) and was incubated 30 min at 37 °C on a shaker. The colon pieces were dissociated using gentleMACS™ Octo Dissociator (Miltenyi pre-defined program: m_intestine_01, Miltenyi Biotec). Incubation and shredding procedure were repeated once more. The colon homogenate was filtered through a 100 μM cell strainer and subjected to gradient centrifugation as described for SI LP cell isolation.

To obtain BM cells, femur and tibia from one hind leg were dissected. Bones were cleaned and ground in a mortar in 3% FBS in 1x PBS. Cells were pelleted, incubated in erythrocyte lysis buffer for 2 min at room temperature and washed in 3% FBS in PBS.

### Fluorescence-activated cell sorting (FACS) and flow cytometry

FcγRII/III monoclonal Ab (clone 2.4G2^48^, purified cell supernatant) was used to block unspecific antibody-binding. Cells were stained with fluorochrome-conjugated or biotinylated Abs in 3% FCS in 1x PBS for 30 min at 4 °C. To detect biotinylated Abs, cells were incubated with fluorochrome-conjugated streptavidin for 20 min at 4 °C. Unless otherwise indicated the following lineage (lin) cocktails of antibodies were used for flow cytometry: CD3, CD8, CD11c, CD19, B220, Gr-1, TCRβ, TCRγδ, Ter-119, and for sorting of ILC3: CD3, CD8, CD11c, CD19, B220, Gr-1, TCRβ, TCRγδ, Ter-119, and NK1.1. Dead cells were stained with LIVE/DEAD^®^ Fixable Aqua Dead Cell Stain kit (Molecular Probes) according to the manufacturer’s protocol.

For the detection of intracellular cytokines, cells were incubated with 10 μg/ml Brefeldin A in complete IMDM medium supplemented with 5% FBS at 37 °C for 2–4 h prior to surface staining and staining with LIVE/DEAD^®^ Fixable Aqua Dead Cell Stain kit. Cells were fixed in 4% paraformaldehyde in PBS for 10 min at room temperature and washed twice in permeabilization solution (0.5% Saponin, 0.01% sodium azide, and 3% FBS in PBS). Cytokines were stained with antibodies diluted in permeabilization solution at 4 °C.

Transcription factor staining was performed using Foxp3 Transcription Factor Staining Buffer Set (eBioscience) according to the manufacturer’s protocol.

For phosphoFlow analysis, sort-purified cells were cultured in complete IMDM medium supplemented with 5% FBS and 20 ng/ml IL-23/IL-1β. After 17 h, cells were washed in PBS and stained with LIVE/DEAD^®^ Fixable Aqua Dead Cell Stain kit supplemented with 20 ng/ml IL-23/IL-1β. After washing, cells were immediately fixed with 2% PFA in PBS for 10 min at 37 °C. After two wash steps, cells were resuspended in BD Perm Buffer III (pre-chilled to −20 °C) and permeabilized 30 min on ice. Cells were washed twice and stained with Abs for 30 min on ice. After two final wash steps, cells were analyzed immediately.

Cell acquisition was performed with FACS Fortessa (BD Biosciences) and data analysis with FlowJo 10 software (Tree Star).

Cells were sorted with FACS Aria (BD Biosciences). Purity of isolated cells was ≥98%.

### In vitro stimulation and cell culture

Sorted Lin^−^CD90.2^+^KLRG1^−^ ILC3s, SI LP, or cLP cells were cultured in complete IMDM medium supplemented with 5% FBS at 37 °C under 95% humidity and with 5% CO_2_. Cells were treated with 20 ng/ml IL-23 and 20 ng/ml IL-1β where indicated. mTORC1 inhibitor rapamycin and mTOR inhibitor PP242 was added to a final concentration of 10 nM and 1 μM, respectively.

### Bone marrow chimeras

*Rag2*^*−/−*^ recipient mice heterozygous for the congenic marker Ly5.1 and Ly5.2 (*Rag2*^*−/−*^*Ly5.1/Ly5.2* mice) were lethally irradiated with two doses of 450 rad from a γ-source (Cs 137 source) with a resting period of 4 h.

Donor *Rag2*^*−/−*^*Ly5.1* and knockout or control mice (*Ly5.2*) were sacrificed. Bones from both hind legs were removed, cleaned, and grounded in a mortar containing DMEM. BM cells were filtered through a 70 μm cell strainer, centrifuged (1400 rpm, 10 min) and subjected to erythrocyte lysis as described above. Cells were washed twice in PBS and filtered through a 40 μm strainer. The donor cells from *Rag2*^*−/−*^ *Ly5.1* mice and knockout or control mice were mixed in a 1:1 ratio. One day after irradiation recipient mice were injected with 1.5 × 10^7^ donor cells/200 μl into the tail vein.

Recipient mice received Baytril (570 mg/l drinking water = 85 mg/kg bodyweight) during the first 10 days. Mice were sacrificed 5–10 weeks after experiment initiation.

### IL-2/α-IL-2 complex treatment

IL-2/α-IL-2 complexes were previously described to induce proliferation of T cells and NK cells in vivo in mice after intraperitoneally (i.p.) injection^30,31^. Complexes were prepared by mixing 2.5 μg recombinant murine IL-2 (0.67 mg/ml) and 7.5 μg anti-IL-2 mAb JES6-1A12 (3 mg ml, BioXCell) per mouse. The IL-2/α-IL-2 mixture was incubated at 37 °C for 30 min, adjusted to 200 μl/mouse with sterile PBS and injected into mice. Mice were sacrificed 1 day post injection.

### α-CD40 Ab-induced colitis model

α-CD40 Ab (Clone FGK45.5) was diluted to 1 mg/ml in sterile PBS. Mice were weighed and i.p. injected with 140 μg α-CD40 Ab^18^. Weight and health condition of mice was monitored daily.

### Rapamycin treatment

For in vivo use, lyophilized rapamycin (LC Laboratories) was resuspended to 250 μg/ml in sterile PBS supplemented with 0.125% dimethyl sulfoxide (DMSO) and 2.5% ethanol. Four hour after α-CD40 Ab treatment, mice were i.p. injected with mock solution (PBS with 0.125% DMSO and 2.5% ethanol) or 2.5 mg/kg rapamycin. Injections were repeated every other day until mice were sacrificed.

### Hematoxylin and eosin staining and histological colitis scoring

Whole cecum and 25% of the proximal colon were extracted, cleaned with PBS and fixed in 4% paraformaldehyde (histology grade, Roth) for 48–72 h. Sample dehydration, paraffine-embedding, and hematoxylin and eosin staining were performed according to standard protocols. Histology colitis scores (0–12) were determined in a blinded fashion by a pathologist. Biopsies were scored by assessing (1) epithelial hyperplasia and goblet cell depletion (none = 0, 1.5× of normal length and/or 25% loss of goblet cells = 1, 2–3× of normal length and/or 25–50% loss of goblet cells = 2, >3× of normal length and/or >50% loss of goblet cells = 3), (2) *LP* inflammatory infiltration (none = 0, some leukocytes at villi tips or lymphoid follicles = 1, marked leukocyte infiltrate and crypt broadening = 2, dense leukocyte infiltrate = 3), (3) proportion of affected area (none = 0, <25% = 1, 25–50% = 2, >50% = 3), and (4) severity markers (none = 0; mild submucosal inflammation or <5 crypt abscesses = 1; mild submucosal inflammation and <5 crypt abscesses = 2; severe submucosal infiltration, >5 crypt abscesses or crypt branching = 2; severe submucosal inflammation and >5 crypt abscesses or crypt branching = 3; ulceration or fibrosis = 3). A total histology colitis score, ranging from 0 to 12 was obtained by summing the four parameter scores.

### Analysis of cytokines in culture supernatants

Cell culture supernatants were collected at the time points indicated and stored at −80 °C. To determine cytokines in cell culture supernatants, LEGENDplex Multi-Analyte Flow Assay Kit Mouse Th Cytokine Panel (13-plex) (Biolegend) was used according to the manufacturer’s protocol. In short, samples or cytokine standards were mixed with assay buffer, capture antibodies, and detection antibodies and incubated on a plate rocker for 2 h at room temperature in the dark. Streptavidin-PE was added and the plate was incubated 30 min at room temperature in the dark. Beads were centrifuged, washed once with wash buffer, and resuspended in wash buffer. Samples were acquired with Cytoflex S and CytExpert Software and analyzed with LEGENDplex V8.0 Software (VigeneTech). Cytokine concentration was normalized to cell numbers.

### Lipocalin-2 enzyme-linked immunosorbent assay

Fecal samples were diluted in sterile PBS to a final concentration of 0.1 g of feces per 1 ml, incubated for 15 min at room temperature, and disrupted manually. Solid fibers were removed by centrifugation (13,000 g, 3 min). Feces supernatant were stored at −20 °C.

Mouse LPC2/NGAL DuoSet ELISA Kit (R&D Systems) was used according to the manufacturer’s protocol. Optical density at 450 nm was measured using SpectraMax 340 microplate reader (Molecular Devices). Wavelength correction was performed by subtraction of optical density measured at 570 nm.

### Statistical analysis

Prism 8 Software (GraphPad Software, Inc) was used to analyze the normality of data sets and statistical significance. Non-normal distributed data were analyzed with Mann–Whitney U test. For normal distributed data sets two-tailed unpaired *t* test or one-way ANOVA with multiple comparison (Bonferroni) were used. Data are shown as mean values ± SD.

## Supplementary information

Supplementary Information
