# Supplementary material for: mTOR signaling mediates ILC3-driven immunopathology
Source: Mucosal Immunol. 2021 Aug 2;14(6):1323–34. doi: 10.1038/s41385-021-00432-4 (PMC8528695; doi:10.1038/s41385-021-00432-4)
Supplement: Supplementary file 1 — Supplementary Information [file 41385_2021_432_MOESM1_ESM.pdf]

Supplementary Figures

mTOR signaling mediates ILC3-driven immunopathology

Claudia Teufel, Edit Horvath, Annick Peter, Caner Ercan, Salvatore Piscuoglio, Michael N. Hall, Daniela Finke, Frank M. Lehmann.

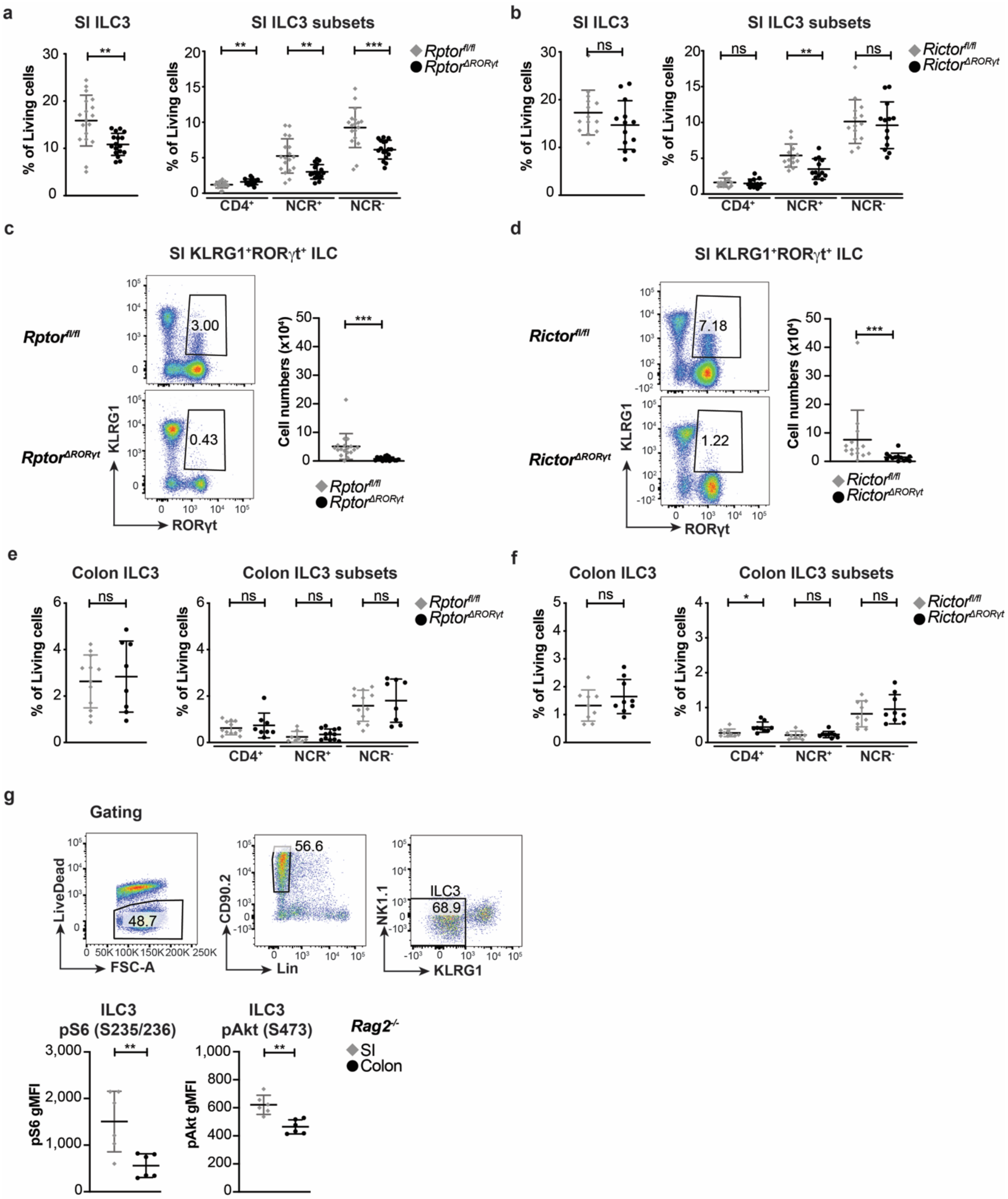

Supplementary Figure 1. ILC subsets in *Rptor*$^{\Delta ROR\gamma t}$ and *Rictor*$^{\Delta ROR\gamma t}$ mice and phosphorylation of mTOR targets in ILC3s.

Cells were isolated from SI LP and cLP of *Rptor*$^{\Delta ROR\gamma t}$ **(a, c, e)** or *Rictor*$^{\Delta ROR\gamma t}$ **(b, d, f)** mice and age-matched *Cre*$^-$ littermates. **(a, b)** SI isolates were analyzed for percentage of total ILC3s and percentage of CD4$^+$, NCR$^-$ and NCR$^+$ ILC3 subsets. ILC3s were gated as shown in Figure 1. *n* = 13-18, 6-8 independent experiments. **(c, d)** SI isolates were analyzed for cell number of RORγt$^+$KLRG1$^+$ ILCs. Dot plots were pre-gated on living CD90$^+$Lin$^-$ cells as shown in Figure 1a. *n* = 13-18, 6-8 independent experiments. **(e, f)** Colonic isolates were analyzed for percentage of total ILC3s and percentage of CD4$^+$, NCR$^-$ and NCR$^+$ ILC3 subsets. ILC3s were gated as shown in Figure 1. *n* = 8-12, 4 independent experiments. **(g)** SI and cLP cells of *Rag2*$^{-/-}$ mice were isolated and phosphorylation of S6 protein and Akt was determined by phospho flow analysis. Indicated values represent the geometric mean fluorescence intensity. ILC3s were gated as depicted. Lin: CD3, CD8, CD11c, CD19, B220, Gr-1, TCRβ, TCRγδ, Ter-119. *n* = 6 mice of 2 independent experiments. ns, not significant; *p≤0.05; **p≤0.01;

***p≤0.001, calculated with two-tailed paired or unpaired Student's t test or Mann-Whitney U test. Bars represent mean ± SD.

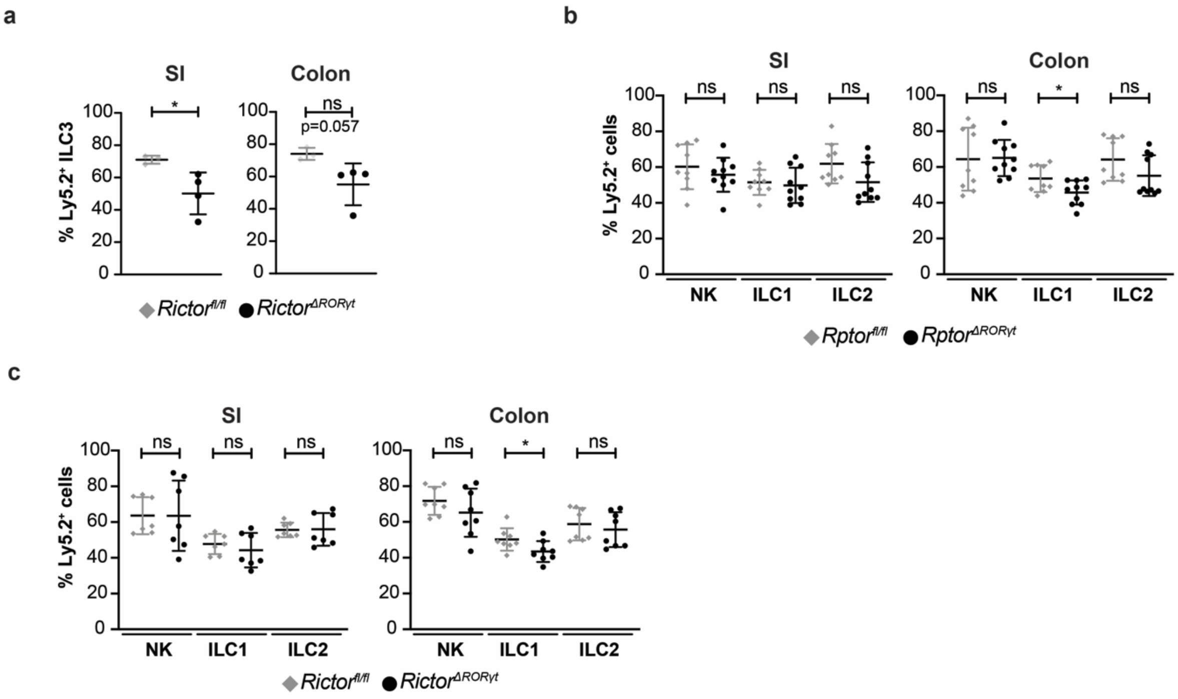

Supplementary Figure 2. Percentage of ILCs in bone marrow chimeras.

(a) Bone marrow chimera model: BM from *Rag2*$^{-/-}$*Ly5.1*$^{+}$ mice was mixed in a 1:1 ratio with BM from *Rictor*$^{\Delta ROR\gamma t}$ mice or *Cre*$^{-}$ littermates, respectively. 10 weeks after transplantation, mice were sacrificed. Cells were extracted from the SI LP and the cLP. Percentage of Ly5.2^{+}Ly5.1^{-} ILC3s within donor-derived cells was determined by flow cytometry. Cells were gated as shown in Figure 2. *n* = 3-4 mice. **(b, c)** Bone marrow chimera model: BM from *Rag2*$^{-/-}$*Ly5.1*$^{+}$ mice was mixed in a 1:1 ratio with BM from either *Rptor*$^{\Delta ROR\gamma t}$ mice **(b)**, *Rictor*$^{\Delta ROR\gamma t}$ **(c)** mice or *Cre*$^{-}$ littermates, respectively. Five weeks after transplantation, mice were sacrificed. Cells were extracted from the SI LP and the cLP. Percentage of Ly5.2^{+}Ly5.1^{-} NK cells (Lin^{-}CD90.2^{+}T-bet^{+}Eomesodermin^{+}), ILC1s (Lin^{-}CD90.2^{+}T-bet^{+}Eomesodermin^{-}RORγt^{-}) and ILC2s (Lin^{-}CD90.2^{+}KLRG1^{+}CD25^{+}CD127$^{+/low}$RORγt^{-}) within donor-derived cells was determined by flow cytometry. Lin: CD3, CD8, CD11c, CD19, B220, Gr-1, NK1.1, TCRβ, TCR$\gamma\delta$, Ter-119. *n* = 8-10 mice from 2-3 independent experiments. ns, not significant; *p ≤ 0.05, calculated with two-tailed unpaired Student's t test or Mann-Whitney U test. Bars represent mean ± SD.

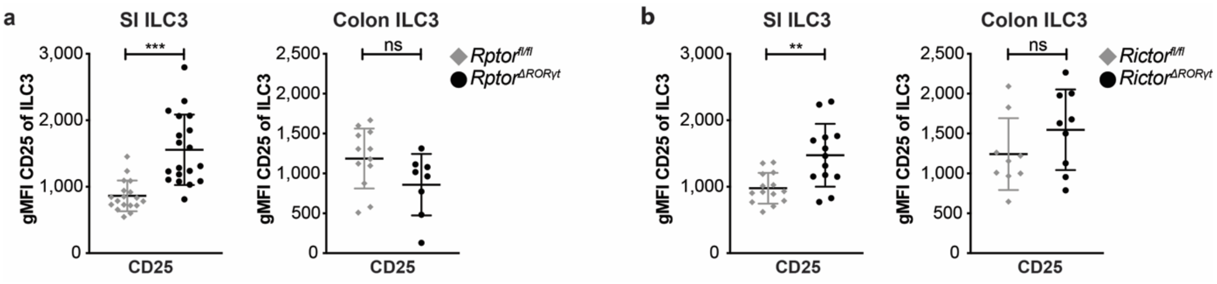

Supplementary Figure 3. CD25 expression in SI and colonic ILC3s derived from *Rptor*$^{\Delta ROR\gamma t}$ and *Rictor*$^{\Delta ROR\gamma t}$ mice.

Cells were isolated from the SI LP and cLP of *Rptor*$^{\Delta ROR\gamma t}$ **(a)** or *Rictor*$^{\Delta ROR\gamma t}$ **(b)** mice and age-matched *Cre*$^{-}$ littermates. CD25 surface expression by ILC3s was determined by flow cytometry. ILC3s were gated as shown in Figure 1. *n* = 12-19, 4-8 independent experiments. ns, not significant; **p≤0.01; ***p≤0.001, calculated with two-tailed unpaired Student's t test. Bars represent mean ± SD.

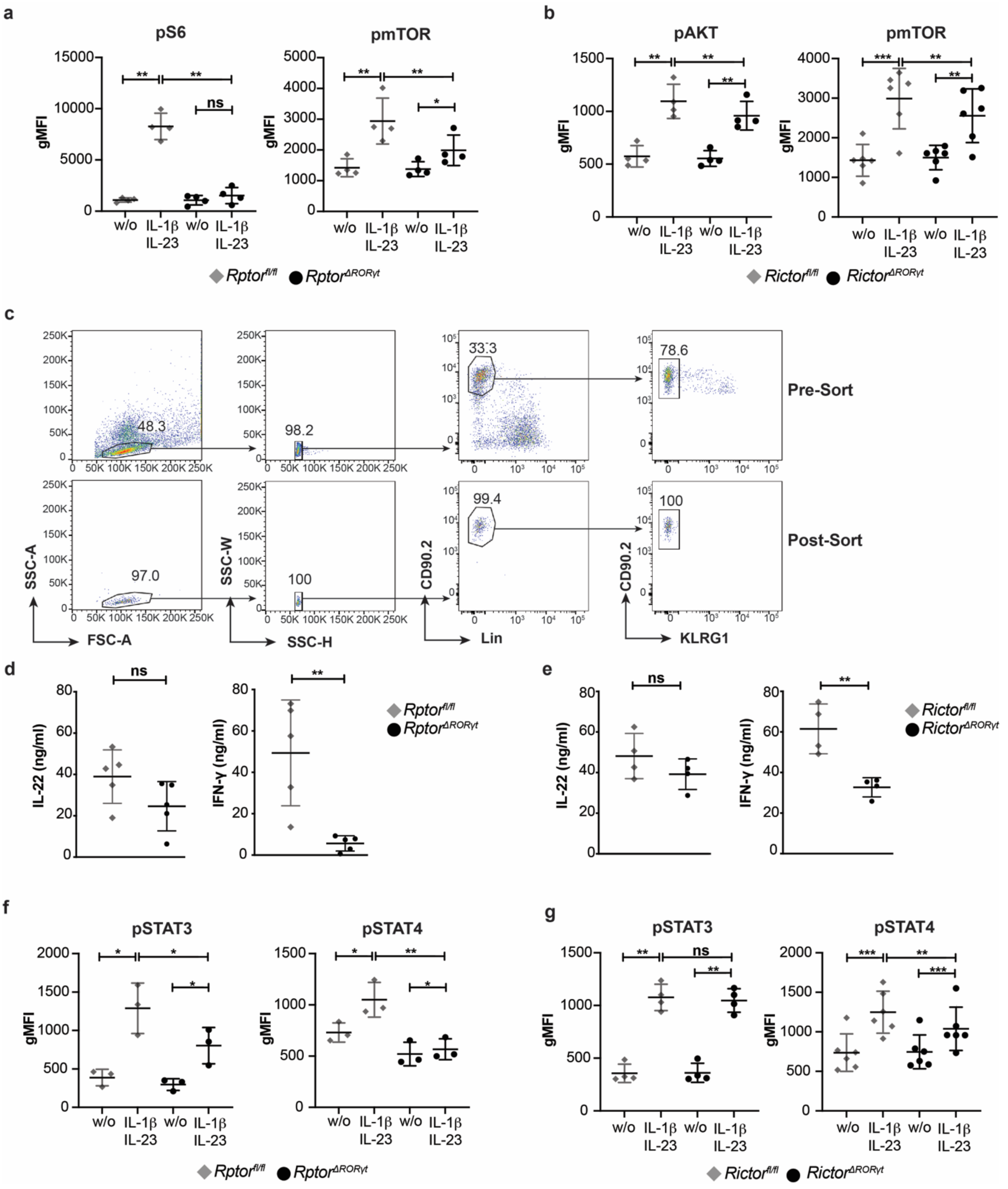

Supplementary Figure 4. Cytokine expression by ILC3s after *in vitro* activation

(a, b) $Lin^-CD90.2^+KLRG1^-$ ILC3s were sorted from the SI of $Rptor^{\Delta ROR\gamma t}$ **(a)** or $Rictor^{\Delta ROR\gamma t}$ **(b)** mice and control littermates and stimulated with 20 ng/ml IL-23 and IL-1β. Phosphorylation of S6 protein and Akt was determined by phospho flow analysis. Indicated values represent the geometric mean fluorescence intensity. The sorting strategy is depicted in Supplementary Figure 4c. For each experiment, cells from 2-3 mice per group were pooled. *n* = 4-6 independent experiments. **(c)** Sorting strategy for ILC3s. **(d, e)** $Lin^-CD90.2^+KLRG1^-$ ILC3s were sorted from the SI of $Rptor^{\Delta ROR\gamma t}$ **(d)** or $Rictor^{\Delta ROR\gamma t}$ **(e)** mice and control littermates and cultured with 20 ng/ml IL-23 and IL-1β for 2 days. Concentration of IL-22 and IFN-γ in the supernatant. For each experiment, cells from 2-3 mice per group were pooled. *n* = 4-5 independent experiments. **(f, g)** $Lin^-CD90.2^+KLRG1^-$ ILC3s were sorted from the SI of $Rptor^{\Delta ROR\gamma t}$ **(f)** or $Rictor^{\Delta ROR\gamma t}$ **(g)** mice and control littermates and stimulated with 20 ng/ml IL-23 and IL-1β. Phosphorylation of S6 protein and Akt was determined by phospho flow analysis.

Indicated values represent the geometric mean fluorescence intensity. The sorting strategy is depicted in Supplementary Figure 4c. For each experiment, cells from 2-3 mice per group were pooled. *n* = 3-6 independent experiments. ns, not significant; *$p \leq 0.05$; **$p \leq 0.01$; ***$p \leq 0.001$, calculated with two-tailed paired or unpaired Student's t test or Mann-Whitney U test. Bars represent mean $\pm$ SD.

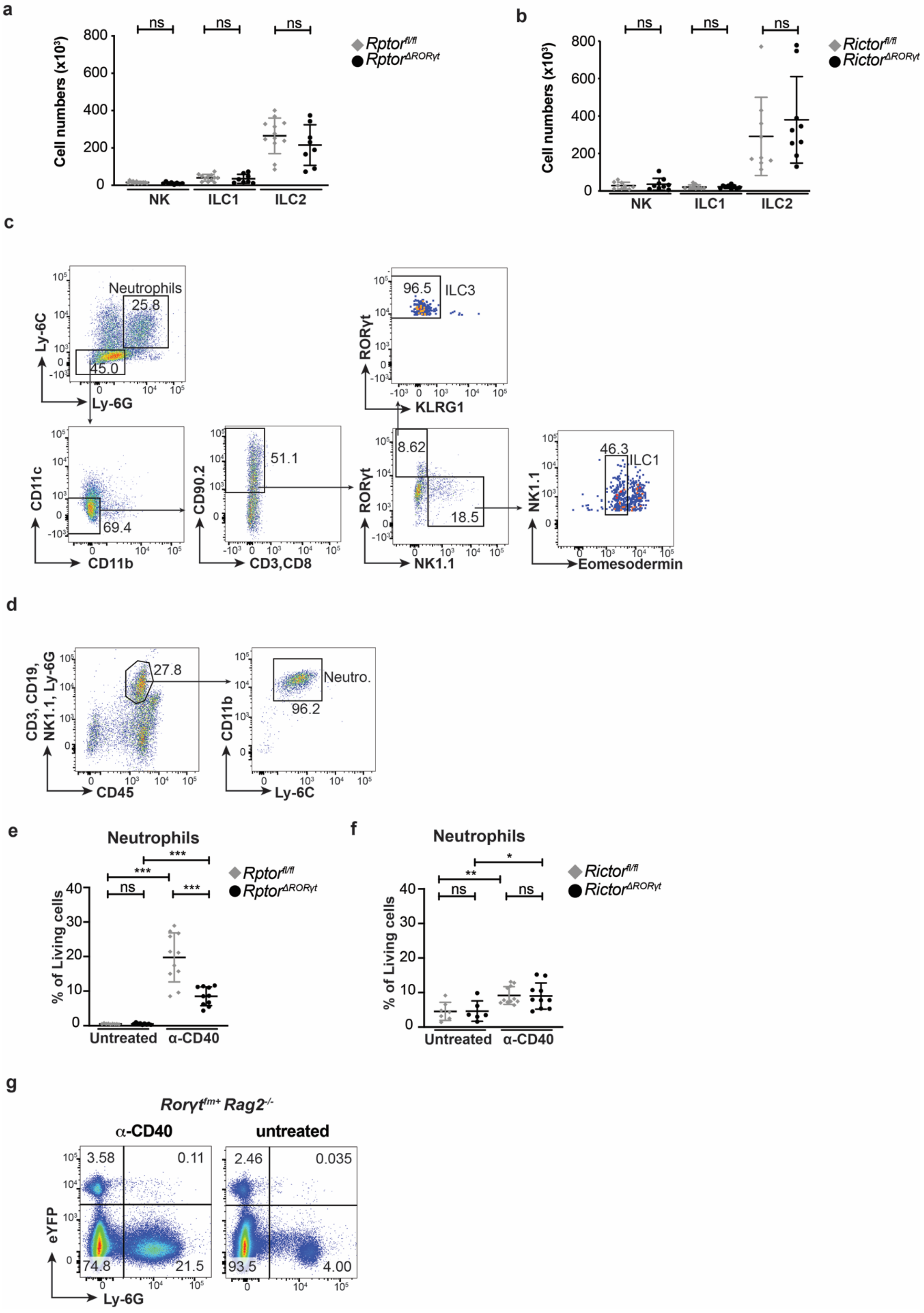

Supplementary Figure 5. Gating strategy and analysis of ILCs and neutrophils in the colon.
(a, b) Total number of colonic NK cells (Lin$^-$CD90.2$^+$KLRG1$^{+/-}$T-bet$^+$Eomesodermin$^+$), colonic ILC1s (Lin$^-$CD90.2$^+$KLRG1$^{+/-}$T-bet$^+$Eomesodermin$^-$RORγt$^-$) and colonic ILC2s (Lin$^-$CD90.2$^+$KLRG1$^+$CD25$^+$CD127$^{+/low}$ RORγt$^-$) in *Rptor*$^{\Delta ROR\gamma t}$ **(a)** or *Rictor*$^{\Delta ROR\gamma t}$ **(b)** mice and age-matched *Cre*$^-$ littermates. Lin: CD3, CD8, CD11c, CD19, B220, Gr-1, TCRβ, TCRγδ, Ter-119. n = 8-17 mice of 4-8 independent experiments. **(c)** Exemplary gating of neutrophils, ILC1

and ILC3 from colonic LP cells cultured overnight for Figures 6c-f,i and j. Cells were gated on living cells. **(d)** Exemplary gating of neutrophils for Figure 6g and h. Cells were gated on living cells. **(e)** *Rptor*$^{\Delta ROR\gamma t}$ or *Rictor*$^{\Delta ROR\gamma t}$ **(f)** mice and age-matched littermates were left untreated or received a single dose of 140 μg α-CD40 Ab. Mice were sacrificed 3 days post-injection. Colonic neutrophils were analyzed by flow cytometry. Neutrophils were gated as depicted in Figure S6b. **(e, f)** Percentage of neutrophils within living cells. *n* = 6-11 mice from of 3-4 independent experiments. **(g)** *Ror*γt^{fm+} *Rag2*$^{-/-}$ mice were left untreated or received a single dose of 140 μg α-CD40 Ab. Mice were sacrificed 3 days post-injection. Colonic cells were analyzed by flow cytometry for eYFP and Ly-6G. Cells were gated on living cells. Representative dot plots of 3 mice per group. ns, not significant; *p≤0.05; **p≤0.01; ***p≤0.001, calculated with two-tailed unpaired Student's t test or Mann-Whitney U test. Bars represent mean ± SD.
